# Fat forward: Cultivating bovine adipocytes on bioscaffolds

**DOI:** 10.1016/j.crfs.2025.101047

**Published:** 2025-04-02

**Authors:** Apeksha Bharatgiri Goswami, Joanna M. Biazik, Johannes le Coutre

**Affiliations:** a School of Chemical Engineering, University of New South Wales, Sydney, New South Wales, Sydney, Australia; b Electron Microscope Unit, Mark Wainwright Analytical Centre, University of New South Wales, Sydney, New South Wales, Sydney, Australia; c Australian Human Rights Institute, University of New South Wales, Sydney, New South Wales, Sydney, Australia

**Keywords:** Bioscaffold, Oats, Buckwheat, Cultured meat, Bovine adipocytes

## Abstract

The advancement of cellular agriculture hinges on replicating the mouthfeel, taste, and texture of conventional meat, which are largely determined by fat tissue composed of adipocytes. However, growing cells at scale remains a significant challenge for the field. This study explores the use of edible bioscaffolds to support the large-scale production of bovine adipocytes.

Scaffold-based approaches are commonly used to facilitate the proliferation of adherent cells within bioreactors, yet identifying suitable, edible scaffolds for cultured meat remains an ongoing challenge. Here, we present an efficient approach for screening biological scaffolds and evaluating their suitability for cultured meat production. We assess whole oats and unhulled buckwheat as potential substrates for bovine preadipocyte attachment, proliferation, and differentiation. Our results demonstrate that both grains support cell adhesion and growth; however, with their favourable surface properties, whole oats emerged as a promising natural bioscaffold for cultured food applications, offering both scalability and nutritional benefits.

## Highlights

• Development of a method to screen plant-derived bioscaffolds for cultured meat production.
• Demonstration of whole oats and unhulled buckwheat supporting bovine preadipocyte attachment and growth.
• Analysis via scanning electron microscopy showing that both grains enable cellular monolayer formation.

## Graphical abstract

This study addresses key challenges in cellular agriculture by developing a rapid, food-safe method to evaluate edible bioscaffolds for cultured meat production. Whole oats emerged as a promising, scalable substrate for bovine adipocyte growth, offering a sustainable solution to replicate the taste, texture, and mouthfeel of conventional meat.

**Figure:**
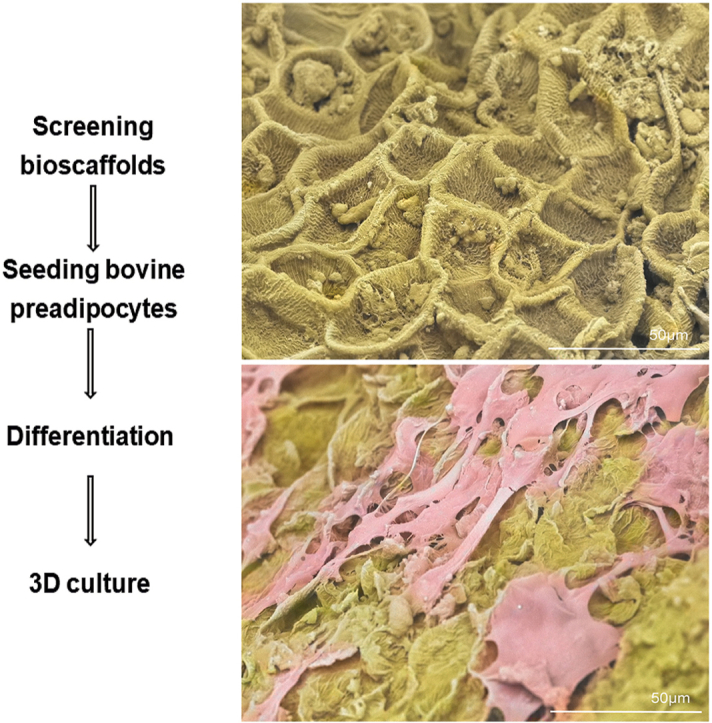

## 1 Introduction

When selecting scaffolds for cultured meat production, choosing the right biomaterial is crucial, as it influences multiple aspects of the final product, such as cell support, useability, safety, sustainability, and scale-up potential (Levi et al., 2022). The biomaterial must facilitate cell attachment and offer a microenvironment that can support cell expansion, differentiation, and their ability to take up nutrients (Post et al., 2020; Schätzlein and Blaeser, 2022; Moslemy et al., 2023). Especially if scaffolds are being designed for the purpose of cultivating cellular agriculture (cellAg) products, they should be compatible with meat-related cell types, such as adipocytes, myocytes or fibroblasts (Post et al., 2020). While muscle cells are essential for cultured meat production, fat cells play a critical role in determining texture, mouthfeel, and flavour. Therefore, studying adipocytes in the context of bioscaffolds is crucial for developing a well-structured and palatable cultured meat product. Additionally, to work well as substitutes for the extracellular matrix (ECM) in a laboratory, scaffolds should mimic the characteristics and components of ECM and the environment of the target tissues (Reiss et al., 2021). Due to their natural bioactivity and compatibility, animal-derived decellularised scaffolds are critical for regenerative medicine, tissue engineering, and cultured meat production (Lu et al., 2022). They retain ECM components that provide structural support, growth factors, and a conducive microenvironment for cell behaviour and differentiation, surpassing synthetic scaffolds in replicating tissue function. However, challenges include high costs, immunogenicity, and scalability for industrial applications. Moreover, to position cultured meat as 100 % animal-free, the scaffold should not be sourced from animal origin. Preparation methods for decellularised scaffolds generally involve a combination of physical, chemical, and biological treatments designed to remove cellular components while preserving the structure of the ECM (Zhang et al., 2022). The scaffold biomaterial in cultured meat should be edible and safe as an ingredient in a food product. Additionally, it is important to pay close attention to the different stages of biomaterial processing to reduce the presence of inedible or harmful substances, including solvents and crosslinkers (Levi et al., 2022).

Alternatively, plant-derived scaffolds provide an eco-friendly alternative by utilising cellulose-rich structures that enhance biocompatibility and reduce costs. While these scaffolds demonstrate considerable potential for *in vitro* tissue culture and facilitating cell alignment, challenges related to reproducibility and large-scale implementation still need to be addressed. Decellularisation of plant materials exploits the naturally present vascular networks for diverse tissue engineering uses (Roy et al., 2023). For instance, primary bovine cells have been cultured on decellularised spinach leaves (Jones et al., 2021), and bovine skeletal cells on broccoli florets (Thyden et al., 2022), primary dog skeletal muscle satellite cells on leaf veins (Luo et al., 2024), and mouse myoblasts on walnut leaf (Rybchyn et al., 2023), show promising physical and nutritional properties for cultured meat applications. Additionally, innovative decellularised plant scaffolds derived from agricultural waste, such as corn husk, demonstrate cell transfer capabilities in dynamic systems and highlight their role in sustainable cultured meat production (Perreault et al., 2023a). Using edible materials in production can improve sensory qualities and eliminate extra separation steps (Nurul Alam et al., 2024). These materials also allow for better control and monitoring compared to traditional scaffolds and bioreactors, resulting in better product quality, consistency, and lower costs (Seo et al., 2023; Andreassen et al., 2022; Perreault et al., 2023b; Su et al., 2023; Suntornnond et al., 2024; Letcher et al., 2022; Ben-Arye et al., 2020; Zheng et al., 2022). Moreover, this method makes it easy to expand the growth surface by adding new scaffolds to the bioreactor, which helps cells move and populate the new bioscaffolds. Edible scaffolds provide benefits like removing the need for separation processes and increasing production efficiency (Ng and Kurisawa, 2021). These materials are, therefore, regarded as promising for use in cultured meat. Nonetheless, the challenge of inadequate cell adherence may require modifications to the proteins or the addition of substances that enhance cell attachment (Cai et al., 2020). Also, they might change the taste, colour, and texture of the final product (Bodiou et al., 2020).

While collagen is a prime scaffold material (Lin et al., 2019), ethical concerns stemming from the reliance on animal sources raise issues related to animal welfare, dietary restrictions, and sustainability, which have driven the development of recombinant collagen from bacterial, yeast, and plant sources (An et al., 2014; Liu et al., 2008; Stein et al., 2009). However, challenges like post-translational modification remain (Werkmeister and Ramshaw, 2012). Protein-based films such as glutenin and zein show potential for cultured meat due to their adherence, proliferation, and differentiation properties (Xiang et al., 2022). Similarly, textured soy protein supports bovine myoblast adhesion, growth, and maturation (Ben-Arye et al., 2020). Synthetic biodegradable polymers, such as PLA and PLGA, also offer promise as 3D scaffolds, provided they align with cellAg's environmental goals (Langelaan et al., 2010; Seah et al., 2022; Krujatz et al., 2022). Bacterial nanocellulose can support myoblast viability and differentiation, though modifications are needed to improve its biocompatibility (Rybchyn et al., 2021). Edible chitosan-collagen hydrogel microcarriers promote rapid cell proliferation across multiple cell types (Zernov et al., 2022), and peanut protein scaffolds enhance porcine smooth muscle cell growth and ECM production, improving cultured meat quality (Zheng et al., 2022).

Despite these improvements in cell culture techniques, large-scale manufacturing remains a challenge for commercial use (Kumar et al., 2023). There is a high demand for affordable and scalable scaffolds designed for meat-related cells. Even at present, a significant issue is that products derived from animals provide the best suitability and efficiency for cell affinity and attachment. Nonetheless, these products are not compatible with an animal-free approach to cultured meat production (Lanzoni et al., 2022; McKee and Chaudhry, 2017). Also, despite their potential, decellularised scaffolds require advancements to address differentiation control, co-culture integration, texture replication, and automated production for widespread adoption in cultured meat and other fields.

Based on these various potential biomaterials previously researched and their functionality, we set forth to conduct a proof-of-concept study to screen for bioscaffolds that are easy to prepare, safe for consumption, and support cell growth. In this study we explore the potential of oats (*Avena sativa*) and buckwheat (*Fagopyrum esculentum*) to serve as a bioscaffolds for cellAg by evaluating their ability to support the adhesion, viability, and proliferation of bovine preadipocytes.

## 2 Methods

### 2.1 Cell culture

Bovine preadipocytes were isolated from the subcutaneous fat tissue obtained from the exterior hind leg of a Black Angus domesticated male cow (*Bos taurus*). The fat tissue was cleaned (Goswami et al., 2024) and processed using a method previously described (Goswami et al., 2025). The cells used in this study have been expanded from a single colony and characterised using Oil Red O staining, RNA sequencing and protein analysis (Goswami et al., 2025). These cells at passage number 35 were maintained in growth media containing DMEM, 10 % v/v fetal bovine serum, 2 mM L-glutamine, 100U/ml penicillin, and 100 μg/ml streptomycin at 37 °C/5 % CO_2_. To differentiate these cells, media containing free fatty acids (FFA), composed of Elaidic, Oleic, Myristoleic, Palmitoleic, Erucic, and Phytanic fatty acids (at 50 μM each) in complete DMEM-high glucose was used.

### 2.2 Oat and buckwheat bioscaffolds

Whole oats and unhulled buckwheat were obtained from Australian Wheatgrass (NSW, Australia). Dry oats and buckwheat were autoclaved to sterilise and then washed repeatedly for five days in sterile isotonic saline on a rotating platform to leech any excess starch which could be harmful to cells. Oats and buckwheat were washed in 100 % ethanol and dried briefly. Sterile oats were then packed into four individual 50 mL Corning® mini bioreactor tubes (creating a packed bed of 3 cm from the bottom). Three bioreactor tubes were used for cell seeding (triplicates) and one for control. Bovine preadipocytes were seeded at 1 × 10^4^ cells/ml concentration on the packed bed of oats in 10 ml of growth media per tube. The control tube was filled with the same volume of growth media without the cells. Seeding was performed every second day for a week (three seedings were performed) and maintained over 30 days in growth media with media changes every 2–3 days. After day 30, the media were replaced with differentiation media and incubated in all seeded bioreactor tubes for three days. The same procedure was performed for the sterile buckwheat grains.

### 2.3 Cell viability assay

After 33 days, all bioreactor tubes were emptied, and the grains were gently washed thrice with PBS. Three grains were randomly selected from each bioreactor tube (control and seeded) and placed into individual wells of a 96-well plate to measure cell viability through a Cell Titer Blue® (CTB) assay by incubating them in growth media containing 10 % (v/v) CTB dye (Promega, WI, USA) for 4 h at 37 °C with 5 % CO_2_. The supernatant was collected and transferred to a black-well plate, and fluorescence (560 nm excitation/590 nm emission) was measured using a CLARIOstar Plus microplate reader (BMG Labtech, Ortenberg, Germany). GraphPad Prism 10 was used for the statistical analyses (unpaired *t*-test).

### 2.4 Protein quantification assay

To calculate the total protein content of adipocyte culture on the scaffold, a Bicinchoninic Acid (BCA) assay was performed. Protein from all grains in the tube was quantified by randomly selecting five washed grains (both control and differentiated) and placing them in individual wells of a 12-well plate. In total, there were 16 wells, each containing five grains. Cells on these grains were solubilised in PBS containing 1 % v/v TritonX-100 for an hour on a shaker at 37 °C. Afterwards, the suspension from each well was collected in 1.5 ml microcentrifuge tubes vortexed and centrifuged at 10,000×*g* for 10 min. A fixed lysate volume of 200 μl was measured according to the manufacturer's instructions. The absorbance was measured at 562 nm using a CLARIOstar plate reader. GraphPad Prism 10 was used for the statistical analyses (unpaired *t*-test).

### 2.5 Scanning electron microscopy (SEM)

Three PBS-washed grains from the seeded bioreactor tubes and one from the control bioreactor tube were randomly selected and placed into separate wells of a 24-well plate. The samples were first fixed overnight in 2.5 % (w/v) glutaraldehyde in 0.2M sodium phosphate buffer at 4 °C. After fixation, the samples were rinsed with 0.1M sodium phosphate buffer and then fixed again in 1 % osmium tetroxide in 0.2M sodium phosphate buffer for 30 min. They were washed three times with 0.1M sodium phosphate buffer and dehydrated using a graded series of ethanol (30 %, 50 %, 70 %, 80 %, 90 %, and 100 %). Subsequently, the samples were dehydrated with increasing concentrations of hexamethyldisilazane (HMDS) and left to air dry in a 100 % HMDS solution. Finally, the samples were mounted onto platinum-coated SEM stubs and examined using a Hitachi TM 4000 Plus SEM operating at 15 kV.

## 3 Results

### 3.1 Scanning electron microscopy highlights a textured surface of oat bran

**Fig. 1:**
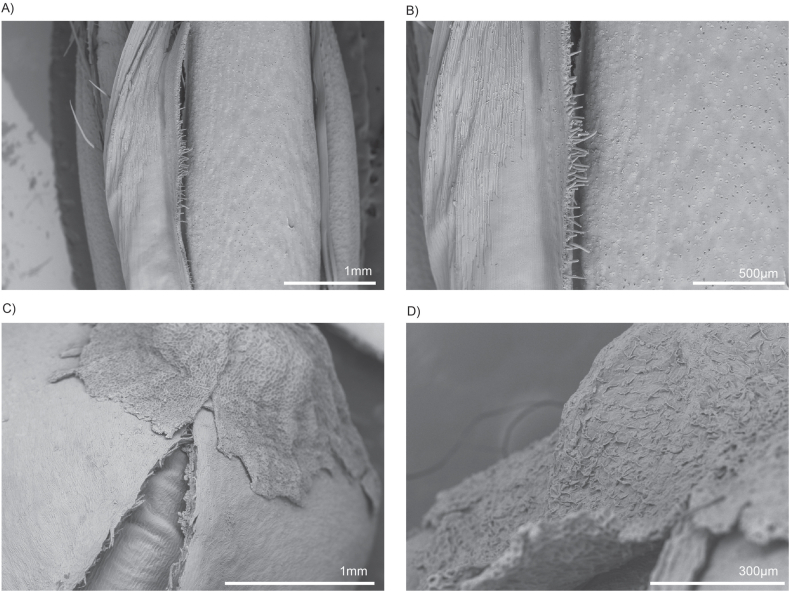
Control SEM images of oat (A–B) and buckwheat (C–D) grains revealed that the outer layers comprise structural features that may promote bovine preadipocyte cell binding. The hull of whole oats has a rough, fibrous texture characterized by ridges and grooves. In contrast, the bran layer beneath it is smoother, with a tightly compacted, fine-grained surface. Unhulled buckwheat grains feature a rugged, granular texture marked by distinct ridges and grooves. The top of the hull displays a naturally grooved and irregular surface, showcasing a pattern of fine ridges, pits, and fibrous structures that enhance its rugged appearance.

SEM analysis revealed that the surface layer of the oat grain bran features a rough, fibrous texture characterised by ridges and grooves. The hull has a coarse, fibrous surface characterised by visible ridges, grooves, and occasional flake-like layers. Its texture is irregular and rough, with a natural matte appearance highlighting the fibrous composition that creates a favourable surface for cell binding. The bran layer beneath the hull is smoother in comparison, with a tightly compacted, fine-grained surface. It shows subtle undulations and a less porous texture, giving it a slightly polished look under the microscope (Fig. 1A–B). Furthermore, the extensive PBS washing performed prior to seeding, which was intended to leach out excess starch, appears to have increased the surface roughness. This complex topography aids in cell attachment and the formation of monolayers and the uneven texture enhances cell attachment by providing multiple contact points and anchorage. The fibrous nature of the grain creates microenvironments suitable to promote adhesion, allowing cells to grip onto the ridges, thus increasing their stability and spreading.

### 3.2 Scanning electron microscopy reveals a grooved surface of buckwheat

Scanning electron micrographs of unhulled buckwheat grains exhibit a rugged, granular texture marked by distinct ridges and grooves. This coarse surface is ideal for cell attachment, as it provides numerous contact points that facilitate adhesion. The top of the hull has a naturally grooved and irregular surface, showcasing a pattern of fine ridges, pits, and fibrous structures that enhance its rugged appearance (Fig. 1C–D). The outer layer appears dense and slightly rough, featuring hard edges alongside subtle depressions. This intricate texture not only improves cell attachment by providing a secure grip but also contributes to the grain's durability, making it a stable substrate for long-term cell culture applications. The structural complexity of the hull creates microenvironments that support cell adhesion and proliferation, resembling features of the natural ECM.

### 3.3 Differentiated bovine adipocytes grow on hull and bran surfaces of whole oat grain

**Fig. 2:**
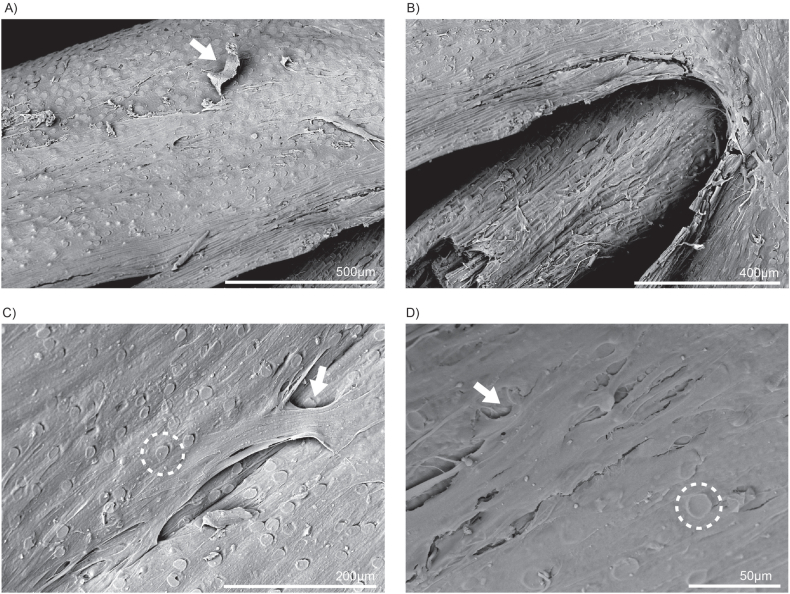
Seeded whole oat grain SEM images (A–D) revealed that differentiated bovine adipocytes actively grow on both the hull and bran layers. The circular ridges (indicated by dotted line) on the bran layer help create a textured surface that allows the cells to attach and expand as a monolayer (indicated by arrows). The monolayer formed by these cells can be seen clearly in A, C, and D.

After three rounds of seeding bovine preadipocytes on washed whole oat grains and maintaining them under differentiation conditions for three days, SEM was conducted to examine cellular attachment and growth. The SEM images provide valuable insights into the behaviour of the cells as they form a monolayer, effectively covering the surfaces of both the grain hull (Fig. 2A–C, and D) and the bran (Fig. 2B). This intricate formation suggests that the cells not only adhere to the surfaces but also organised themselves to enhance their overall functionality. Specifically, the cells exhibit bridge-like connections on the hull, which facilitated expansion and likely played a role in creating a stable environment for further cellular activities (Fig. 2C). Furthermore, it is evident that the textured surfaces of both the hull and bran significantly assist in cell anchorage. The physical characteristics of these surfaces, including their roughness and topography, provide natural points of attachment for the cells, promoting a stronger and more effective adhesion process. This observation confirmed our initial hypothesis regarding the suitability of the microenvironment between the hull and bran layers of the whole oat grain. By creating an optimal setting for cell seeding, the unique features of these layers likely contribute to increased cell viability and activity.

### 3.4 Differentiated bovine adipocytes grew on the surface of the buckwheat hull grain

**Fig. 3:**
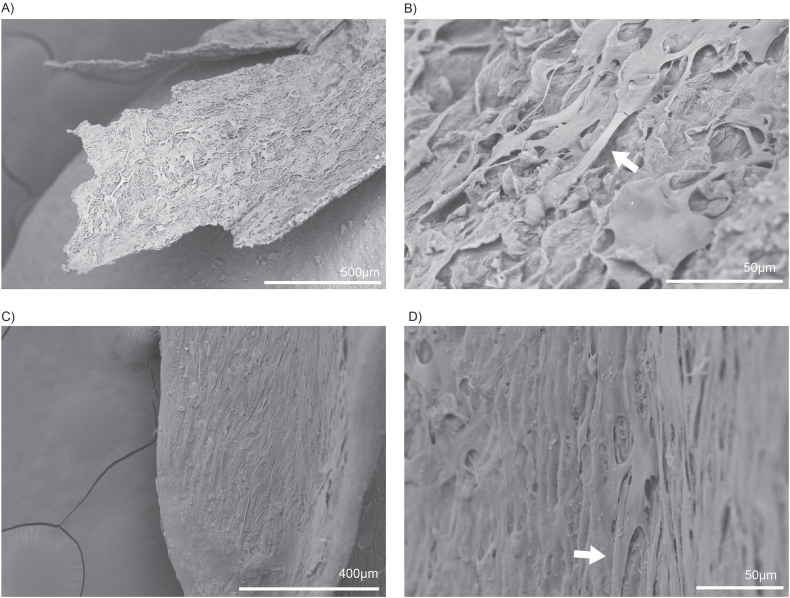
Seeded unhulled buckwheat grain SEM images (A–D) revealed that the differentiated bovine adipocytes actively grow on the outer hull layer. The cells are observed expanding on the upper half of the hull, with a bridge-like structure (indicated by arrows) forming between the cells (A–B). Additionally, the cells expand and form monolayers across the rest of the hull (C–D). A noticeable difference in the textures on the surface can also be seen.

Similar growth was observed on the surface of buckwheat hulls after three rounds of seeding and three days of differentiation of bovine preadipocytes. The cells covered the hull thoroughly, extending from the top (Fig. 3A–B) to the bottom (Fig. 3C–D). This coverage is evidenced by the cell monolayer deposits observed throughout the surfaces. In contrast to oat grains, which possess a tightly packed hull, the structure of the buckwheat hull permits a greater degree of exposure for the cells to interact with its surface. Moreover, there are notable differences between the upper and lower sections of the buckwheat hull. The upper part is characterised by a more fibrous texture, which may provide additional structural support and enhance the attachment of the cells. In contrast, the lower part of the hull features a finely ridged surface, creating a different environment for the cells. Despite these differences in texture, SEM indicated that the cells could expand and thrive equally well on both surfaces, suggesting versatile adaptability in their growth conditions.

### 3.5 Whole oat is a more effective bioscaffold for bovine preadipocytes than unhulled buckwheat

**Fig. 4:**
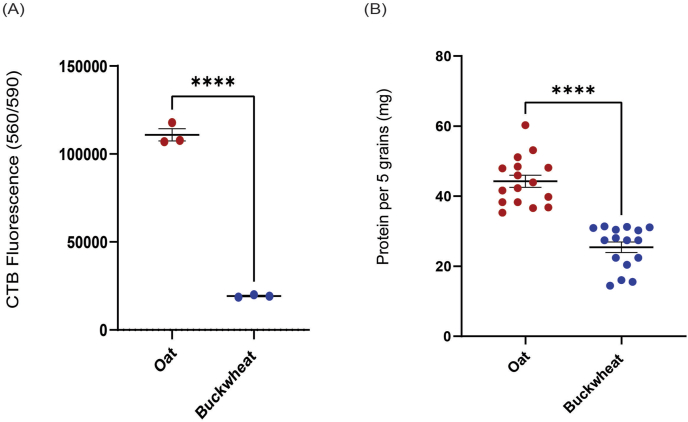
Cell viability and protein quantification assays evaluated the ability of oat and buckwheat grains to support cell attachment and growth. Quantification of viable cells bound to grains showed that whole oats are more efficient bioscaffolds than buckwheat for bovine preadipocytes. Results from whole cell protein quantification performed corroborated these results. Both grains were seeded with bovine preadipocytes (or no cell controls for background quantification). They were maintained under differentiation conditions for 3 days and assessed for bound cellular material by (A) CTB cell viability assay or total cellular protein concentration (B). Statistical significance values shown were determined by unpaired *t*-test.

The seeded grains were further analysed using CTB and BCA assay to confirm our hypothesis and the SEM analysis. Quantification of cells that were retained on both grains was measured using resazurin dye cell viability assay and total cellular protein. Post 30 days of seeding and maintenance under differentiation conditions, individual grains of oats and buckwheat (n = 3) were assessed; oat grains showed significantly higher levels of cell viability by CTB assay compared to buckwheat (Fig. 4A, *p-value* <0.0001).

Simultaneously, protein from all the grains in the tube was quantified by randomly selecting and pooling 5 grains together of oat and buckwheat grains (total n = 80 each), respectively. Whole oat grains again showed increased bound total cellular protein levels than buckwheat (Fig. 4B, *p-value* <0.0001).

## 4 Discussion

This study examined the potential of whole oats and unhulled buckwheat as bioscaffolds for culturing and differentiating bovine preadipocytes. The findings underscore the significant impact of the structural and textural properties of these grains on cell adhesion, viability, and proliferation, providing valuable insights for their application in cellAg.

SEM analysis demonstrated that both grains have complex surface textures that promote cell attachment, although they differ in their structural features and effectiveness as scaffolds. Whole oats possess a rough, fibrous texture with prominent ridges and grooves, creating an intricate topography that enhances cell adhesion. This surface mimics the ECM, offering numerous contact points for anchorage and facilitating cell stability and expansion. By contrast, buckwheat grains feature a rugged, granular surface with distinct ridges, grooves, and fibrous elements. While this texture also aids cell adhesion by providing multiple contact points, the uniformity and density of oat grain surfaces seem to offer a more robust environment conducive to cell proliferation and stability.

The washing protocol employed for both grains included PBS rinses to remove excess starch, further improving their surface parameters. The increased roughness was especially beneficial for whole oats, as the enhanced microenvironment created by their ridges and grooves promoted greater cell adhesion and monolayer formation. Although buckwheat grains also benefit from the washing process, they exhibited less consistent cell attachment, likely due to variations in surface porosity and texture between the upper and lower sections of the hull.

Quantitative analyses supported these observations. When quantified, cells attached on whole oats demonstrated significantly higher cell viability and protein content than buckwheat, as shown by the CTB and BCA assays. The superior performance of oats may be attributed to the synergistic properties of its hull and bran layers. The hull's rugged texture and the bran's smoother yet tightly compacted surface provided complementary conditions that facilitated initial adhesion and sustained cell growth. By contrast, buckwheat grains, despite their coarse surface, showed lower cell retention and protein amount, perhaps due to their less uniform texture and greater porosity, which may have hindered their ability to create consistent microenvironments for cell attachment.

The structural differences between the grains also influenced their long-term suitability for cell culture. Oats provide a more stable and durable substrate, likely due to their dense fibrous composition and interconnected ridges and grooves that enhanced cell anchorage. While buckwheat grains are mechanically sturdy, they may need further optimisation to match the performance of oats. Potential surface treatments or coatings could be explored to improve the uniformity and adhesion properties of buckwheat.

Furthermore, serum was incorporated throughout the process as the study was to provide a proof of concept; further experiments to test the scalability using bioreactors and longer differentiation times will be aimed at performing without using serum. Our laboratory has previously demonstrated an alternate media formulation (Charlesworth et al., 2024). Additionally, we acknowledge that breed-specific variations in fat metabolism exist, and the exclusive use of Black Angus cattle in this study may not fully capture the diversity of bovine adipogenic responses. Metabolic differences between breeds could influence lipid accumulation, cell behaviour, and overall fat tissue functionality in cultured systems. Future studies should investigate adipogenesis across multiple breeds and assess additional cell types to more comprehensively evaluate scaffold efficiency. Examining the scalability of these bioscaffolds in bioreactor systems will be essential to fully exploit their potential in cellAg and tissue engineering.

In conclusion, comparing oat and buckwheat grains highlights the critical role of substrate surface properties in influencing cellular behaviour. Oat grains displayed a superior capacity for supporting bovine preadipocyte culture, indicating their potential as a natural, cost-effective bioscaffold for cellAg. Buckwheat grains, while less effective, still hold promise with targeted modifications. Future research could focus on modifying the surfaces of oat and buckwheat grains and extending the duration of differentiation and culture.

## 5 Concluding remarks

In conclusion, whole oat grains and unhulled buckwheat are effective bioscaffolds for the culture of bovine preadipocytes, with their textured surfaces enhancing cell attachment and growth. SEM analysis indicated that both types of grains supported the formation of cellular monolayers; however, oats exhibited higher cell viability and protein content compared to buckwheat. These findings suggest that whole oats are potentially superior bioscaffolds for long-term cell culture and differentiation, presenting a promising application opportunity in cellAg.

## CRediT authorship contribution statement

**Apeksha Bharatgiri Goswami:** Conceptualization, Writing – original draft, conceptualised, performed experiments and drafted the manuscript. **Joanna M. Biazik:** performed the experiments related to SEM. **Johannes le Coutre:** Conceptualization, Supervision, provided funding, developed the concept, supervised the research, and edited the manuscript.

## Declaration of competing interest

The authors declare that they have no known competing financial interests or personal relationships that could have appeared to influence the work reported in this paper.

## Data Availability

No data was used for the research described in the article.
